# Systematic review and meta-analysis of birth weight and PFNA exposures

**DOI:** 10.1016/j.envres.2023.115357

**Published:** 2023-01-24

**Authors:** J.M. Wright, A.L. Lee, K.M. Rappazzo, H. Ru, E.G. Radke, T.F. Bateson

**Affiliations:** a US EPA, Office of Research and Development, Center for Public Health & Environmental Assessment, Chemical and Pollutant Assessment Division, USA; b US EPA, Office of Research and Development, Center for Public Health & Environmental Assessment, Public Health and Environmental Systems Division, USA

**Keywords:** PFAS, PFNA, Birth weight, Pregnancy, Developmental

## Abstract

We used a systematic review that included risk of bias and study sensitivity analysis to identify 34 studies examining changes in birth weight (BWT) in relation to PFNA biomarker measures (e.g., maternal serum/plasma or umbilical cord samples). We fit a random effects model of the overall pooled estimate and stratified estimates based on sample timing and overall study confidence. We conducted a meta-regression to further examine the impact of gestational age at biomarker sample timing. We detected a −32.9 g (95%CI: −47.0, −18.7) mean BWT deficit per each ln PFNA increase from 27 included studies. We did not detect evidence of publication bias (p_E_ = 0.30) or between-study heterogeneity in the summary estimate (p_Q_ = 0.05; I^2^ = 36%). The twelve *high* confidence studies yielded a smaller pooled effect estimate (β = −28.0 g; 95%CI: −49.0, −6.9) than the ten *medium* (β = −39.0 g; 95%CI: −61.8, −16.3) or four *low* (β = −36.9 g; 95%CI: −82.9, 9.1) confidence studies. The stratum-specific results based on earlier pregnancy sampling periods in 11 studies showed smaller deficits (β = −22.0 g; 95%CI: −40.1, −4.0) compared to 10 mid- and late-pregnancy (β = −44.2 g; 95%CI: −64.8, −23.5) studies and six post-partum studies (β = −42.9 g; 95%CI: −88.0, 2.2). Using estimates of the specific gestational week of sampling, the meta-regression showed results consistent with the categorical sample analysis, in that as gestational age at sampling time increases across these studies, the summary effect estimate of a mean BWT deficit got larger. Overall, we detected mean BWT deficits for PFNA that were larger and more consistent across studies than previous PFAS meta-analyses. Compared to studies with later sampling, BWT deficits were smaller but remained sizeable for even the earliest sampling periods. Contrary to earlier meta-analyses for PFOA and PFOS, BWT deficits that were detected across all strata did not appear to be fully explained by potential bias due to pregnancy hemodynamics from sampling timing differences.

## 1. Introduction

Per-and poly-fluoroalkyl substances (PFAS) represent a difficult risk assessment and risk management challenge given that estimates range from 9,000 to over 12,000 PFAS in production, use, and/or in the environment (Williams et al., 2022). Known as “forever chemicals”, many of these PFAS have very long half-lives, which lead to their persistence in the environment and the human body (Li et al., 2018; Olsen et al., 2007; Chang et al., 2008). PFAS have been associated with various health effects including immunological, hormonal, reproductive, and developmental effects (Radke et al., 2022). Measures of fetal growth restriction, such as low birth weight, are recognized predictors of infant mortality and in utero effects are known to have long-term health effects later in life, as evidenced by Developmental Origins of Health and Disease research (Barker, 1997). It is important to note that many PFAS co-occur and present as a mixture in the environment and lead to ubiquitous exposures in humans. Some associations with developmental effects (such as fetal and childhood growth restriction, spontaneous abortion and gestational duration impacts) have been reported in epidemiological studies for different PFAS, but weight of evidence characterizations are mixed. One source of uncertainty demonstrated in recent meta-analyses of mean birth weight (BWT) (Steenland et al., 2018; Dzierlenga et al., 2020) includes dilution and increased renal filtration changes in perfluorooctanoic acid (PFOA) and perfluorooctanesulfonic acid (PFOS) biomarker levels due to pregnancy hemodynamics (e.g., increased blood plasma volume as a result of decreased mean arterial pressure, increased cardiac output, and systemic vasodilation, Chapman et al., 1998; Sanghavi and Rutherford, 2014; Sagiv et al., 2018) may lead to bias in epidemiological studies—especially those based on samples collected late in pregnancy.

These meta-analyses found that BWT deficits were largely diminished or even null when studies were restricted to earlier sampling periods like trimester one. The meta-regression of PFOS found in Dzierlenga et al., 2020 confirmed these differences across sample timing strata based on a continuous estimate of sample timing. A decrease in PFAS levels has been noted in serial measurements for most PFAS during pregnancy, namely PFOA, PFOS, and perfluorononanoic acid (PFNA) (Glynn et al., 2012; Chen et al., 2021). These hemodynamic changes have been proposed as a potential source of bias for associations between different PFAS and neonatal and early childhood growth measures.

Only one systematic review and meta-analysis for PFNA and mean BWT was found in the literature (Gui et al., 2022), although some methodological differences including a smaller sample size (n = 14 studies) may preclude direct comparisons with our meta-analyses of 27 studies here. As part of an effort to gauge the potential health effects related to PFNA exposure, we examined PFNA exposure in relation to BWT in a meta-analysis and meta-regression. We conducted a systematic review of the published literature and assessed the risk of bias and sensitivity related to eligible studies that met our inclusion criteria. As part of this effort, we assessed statistical heterogeneity between the studies, the potential for publication bias and used sub-group and meta-regression analyses to highlight any differences.

## 2. Methods and materials

### 2.1. Systematic review methods

The systematic review methods used to support this meta-analysis are detailed below and outlined in the Integrated Risk Information System (IRIS) Handbook US EPA, 2022). A systematic review protocol used to guide the development of five IRIS PFAS assessments (including PFNA) was used here after being released for public comment in 2019 and updated in 2021 (US EPA, 2021). The protocol presents the methods for conducting the systematic reviews that were used for the study evaluation in this effort.

### 2.2. Literature search

We used the Populations, Exposures, Comparators, Outcomes (PECO) criteria from the systematic review of multiple PFAS (US EPA, 2021) developed by the IRIS program to guide the literature search screening efforts in support of its hazard and dose-response assessments (Supplemental Table 1) and developed additional study inclusion criteria for this meta-analysis. In short, the database searches entailed use of Boolean search strings (Supplemental Table 2) to identify manuscripts examining PFNA exposures and health effects published prior to July 2022, including: PubMed (National Library of Medicine); Web of Science (Thomson Reuters); Toxline (National Library of Medicine); TSCATS (Toxic Substances Control Act Test Submissions). The literature search queries had no date or language restrictions. In addition, relevant literature not found through database searching was identified by:

- Review of studies cited in any PFNA PECO-relevant studies and published journal reviews; finalized or draft U.S. state, U.S. federal, and international assessments (e.g., the Agency for Toxic Substances and Disease Registry [ATSDR] assessment released publicly in 2021).
- Review of studies submitted to federal regulatory agencies brought to our attention.
- Identification of studies during screening for other PFAS.
- Other gray literature (i.e., primary studies not indexed in typical databases, such as technical reports from government agencies or scientific research groups; unpublished laboratory studies conducted by industry; or working reports/white papers from research groups or committees) brought to our attention.

The Boolean string search terms and number of total studies identified in the IRIS PFNA literature search for each database are provided in Supplemental Table 2. All identified studies, author communication and related records were stored in EPA’s HERO database (https://hero.epa.gov). The literature was screened by two independent reviewers with a process for conflict resolution, first at the title and abstract level and subsequently at the full-text level, using structured forms in DistillerSR (Evidence Partners; https://distillercer.com/products/distillersr-systematic-review-software/). Literature inventories for PECO relevant studies and studies tagged as “potentially relevant supplemental material” during screening were created to facilitate subsequent review of individual studies or sets of studies by topic specific experts. From the comprehensive literature searches and subsequent screening, we restricted the results relevant to this meta-analysis to those with developmental effects.

### 2.3. Inclusion criteria for meta-analysis

Following study evaluation, additional criteria were used to select the observations used here. The meta-analysis inclusion criteria included epidemiological studies which reported beta coefficients (βs), 95% confidence intervals (CIs), standard errors and/or p-values relating mean BWT changes (in grams (g) or kilograms (kg)) in PFNA exposure biomarkers in the overall population or stratum-specific data available for both sexes. These biomarkers include umbilical cord measures as well as maternal serum measured before, during and after pregnancy.

Given our extensive efforts to convert and re-scale data results for enhanced comparability, we evaluated all studies regardless of whether they reported transformed or untransformed exposure data and irrespective of how they expressed their continuous exposure study results. Studies reported changes in mean BWT for different unit result expressions including quantile exposure measures and different continuous scales.

### 2.4. Study evaluation

Details on the study evaluation including potential risk of bias and sensitivity considerations are found in the US EPA PFAS protocol (US EPA, 2021). For each study, at least two independent reviewers evaluated each domain (Participant Selection, Exposure Measurement, Outcome Ascertainment, Confounding, Analysis, Selective Reporting, and Study Sensitivity) and determined a consensus rating specific to each domain and the overall confidence level. Ratings for each domain were either Good, Adequate, Deficient, or Critically Deficient, while overall confidence ratings included *high, medium, low*, or *uninformative*. The overall confidence ratings were based on expert judgment of the likely impact that the identified domain limitations had on the results; no pre-defined weighting of domains or numerical scores were used. Details on the ratings, which reflect a consensus judgment between reviewers, are defined in Supplemental Fig. 1.

### 2.5. Data pre-processing

Following exclusion of *uninformative* studies and those that did not meet the inclusion criteria, data pre-processing for the remaining studies in the meta-analyses occurred. Standard unit conversions were conducted when applicable. For example, we converted BWT results reported in kg to g, and gestational age measures of central tendency and variability (e.g., median, mean, standard deviation, and ranges) reported in days to measures based on weeks. Those studies only reporting sex-specific estimates for boys and girls were pooled individually using inverse-variance weighting which provided an effect estimate for the overall study population.

We rescaled studies that used either log_10_ or log_2_ to natural logarithmic (ln) units (i.e., log_e_). We also converted results that were presented in inter-quartile ranges or units of standard deviation changes into a ln-unit change. To increase the number of available studies, we also converted the studies reporting results in the natural scale (i.e., per ng/mL) into ln units (i.e., per ln(ng/mL)) using a percentile-based re-expression method. This approach developed by Dzierlenga et al., 2020 involved plotting the reported linear function for the main effect using 25th – 75th percentiles at 10 percentile intervals of the exposure distribution in each study and then fitting a ln function to those points. This process was repeated using the reported upper and lower confidence intervals to estimate the bounds of the ln function and thus the estimated standard error of the ln function.

### 2.6. Statistical analysis

All statistical analyses are carried out using the open-source platform, R (Version 4.0.3), and all meta-analytic techniques are carried out using the meta-analysis package, metafor, in R (Viechtbauer, 2010).

### 2.7. Overall meta-analysis

The meta-analysis was based on a random effects model following the assumption that each study produced an estimate of a study-specific true effect that varies across studies (Borenstein et al., 2009). Such between-study variation is possible within a collection of epidemiological studies, due to differences in study designs, populations, or other unknown factors. To closely examine the appropriateness of choosing a random over a fixed effects framework, we carried out a sensitivity test. Inverse-variance weighing was employed to minimize the influence of both sampling variance and between-study variance, i.e., heterogeneity, on the pooled effect estimate. The amount of between-study variance was estimated using restricted maximum likelihood and characterized by two metrics: the I^2^ statistic and Cochrane’s Q Test. The I^2^ statistic represents the percent of variation in the pooled estimate due to between-study heterogeneity. Based on the range of values shown in the Cochrane’s I^2^ guidelines (Higgins et al., 2022), we considered I^2^ statistics <40% to represent *low* heterogeneity, with values from 40 to 69% being *moderate*, and values ≥ 70% to represent *high* heterogeneity. Cochrane’s Q test evaluates whether the dispersion of study-specific estimates about the pooled effect estimate is statistically significant via a p-value (p_Q_), based on significance level (α) of 0.05. These tests may be prone to low statistical power when few studies are available, potentially complicating interpretation of the examinations of heterogeneity. Thus, consideration of both statistics in conjunction is recommended to identify situations where heterogeneity may be present (Huedo-Medina et al., 2006). Studies that contributed most to overall heterogeneity were identified using a leave-one-out analysis. Publication bias was evaluated visually using funnel plot asymmetry (Fodor et al., 2018) and the Egger’s regression test (Egger et al., 1997; Higgins et al., 2003) based on an alpha level (p_E_) of 0.05.

### 2.8. Stratified analyses

We employed stratified analyses to evaluate whether the pooled effect estimate varied by study confidence or by the timing of maternal serum sampling. All stratified meta-analyses were carried out using the *metafor* package in R (Version 4.0.3). We fit separate random-effects models for each stratum, producing estimates that account for possible heterogeneity among studies. A fixed effects model was also used to test for statistically significant differences across the subgroups (Borenstein et al., 2009). A p-value less than 0.05 from this hypothesis test is indicative of no differences between any of the strata. Strata-specific statistical tests conducted on subgroups with lower sample sizes are subject to lower power, susceptible to higher uncertainty and, therefore, should be interpreted with caution.

To examine the potential impact of the timing of biomarker collection, studies were assigned to sample timing strata based on reported trimesters of sampling as well as sampling ranges or interquartile ranges or measures of centrality when measures of spread were unavailable (see Supplemental Table 3 for details on sample timing distributions and strata assignments). To assess the impact of sample timing, we employed a three-strata approach with subgroups comprised of samples collected earlier in pregnancy, mid-to late-pregnancy, and post-pregnancy. Earlier pregnancy included studies reporting samples from pre-conception (0 days), the first trimester (0 days to13 weeks and 6 days) or a combination of the first and second trimesters (0 days to 27 weeks and 6 days); mid-to late-pregnancy studies exclusively sampled in the second trimester (14 weeks and 0 days to 27 weeks and 6 days), a combination of the second and third trimester (14 weeks and 0 days to birth), or the third trimester only (28 weeks and 0 days to birth); post-pregnancy studies sampled at or after birth (American College of Obstetrics and Gynecology, 2022).

### 2.9. Meta-regression

To further examine the impact of biomarker sample timing beyond categorical groupings, we conducted a meta-regression based on continuous gestational age at sampling using measures of central tendency for each study. We used a variety of approaches to develop centrality estimates of gestational age if these values were not reported by study authors. Typically reported measures of centrality included medians, means, and midpoints. In the absence of these values, we calculated midpoints of ranges, midpoints of trimesters (for the minimum of Trimester 1, we used 6 weeks to account for delayed pregnancy detection; for the maximum of Trimester 3, we used average age at birth or, if not reported, 40 weeks), weighted mean of means, weighted mean of midpoints, or weighted mean of medians (see Supplemental Table 3 for details on calculations). In situations where more than one measure of centrality is reported, we preferred medians over means to account for possible skewness and means over midpoints. For umbilical cord blood samples, we used the mean gestational age at birth as the centrality estimate. For those umbilical cord studies that did not report these values an average delivery date of 40 weeks was used to estimate the mean sampling value at birth.

### 2.10. Sensitivity and additional analyses

In addition to the primary, three-strata approach to examining sample timing, we examined an alternate strategy of collapsing the groups, early, mid-to late- and post-pregnancy, into two groups. This alternative two-strata approach used the same definition of earlier pregnancy but combined the mid-to late- and post-pregnancy into a single stratum (i.e., Late + Post). Given the potential for error that may arise from re-expressions (Linakis et al., 2021; Dzierlenga et al., 2020), we examined the robustness of the results to re-expression which allowed additional studies reporting results on the natural scale to be included and compared directly. Another minor source of uncertainty we examined via sensitivity analysis, was a lack of clarification on the type of log transformation used in Kwon et al. (2016). Thus, we examined the impact of the ln-unit assumption of their results in our main pooled analyses in comparison to results based on log_2_ and log_10_. We also included an analysis of early and mid/late biomarker sampling amongs the *high* confidence studies. Lastly, we included an additional analysis of fixed effect models for comparison to the random effects models presented here.

## 3. Results

There were 357 human health effect studies identified that met the PECO criteria of which 65 were developmental effects studies. Ten additional studies were identified though reference screening and author knowledge, giving a total of 75 developmental studies identified through title-abstract and full text screening. From this subset of developmental effect studies, we identified 41 observational epidemiologic studies of PFNA that examined mean BWT changes which allowed for study evaluation (Supplemental Fig. 2).

Among these 41 studies, all but one (Hall et al., 2022) reported data on birth weight differences in relation to PFNA exposures based on maternal on infant blood serum or plasma. Four studies reporting only categorical data were not included in the meta-analysis (Cao et al., 2018; Eick et al., 2020; Gao et al., 2022; Hall et al., 2022). Two of these studies did not detect BWT deficits across PFNA tertiles (Cao et al., 2018; Eick et al., 2020), while two reported some deficits that varied across quartiles and sex (Gao et al., 2022; Hall et al., 2022). Given demonstrated heterogeneity in BWT results across sexes in the PFAS literature, we also excluded a study in boys only (Marks et al., 2019) which showed large deficits (β = −169.6 g; 95%CI: −448.3, 109.2) per each ng/ml increase in PFNA and evidence of an exposure-response relationship across categorical exposures. To avoid duplication, we restricted the meta-analysis to the larger study population where multiple publications reported results from the same birth cohorts (i.e., overlapping study populations were not double counted). For example, the Rokoff et al. (2018) study overlapped with the Project Viva study by Sagiv et al. (2018), as did the Bjerregaard-Olesen et al., 2019 study with Aarhus birth cohort detailed in Bach et al. (2016). Similarly, the Woods et al. (2017) study overlapped the Shoaff et al. (2018) from the Health Outcomes and Measures of the Environment cohort. Three studies (Kishi et al., 2015; Kobayashi et al., 2017; Minatoya et al., 2017) were also not considered further, because they had overlapping data from the Hokkaido Study on Environment and Children’s Health birth cohort population detailed in Kashino et al. (2020). After the few exclusions above and limiting the analyses of the same cohorts to these six primary studies, 30 non-overlapping studies that met the inclusion criteria and had mean BWT data in the overall population or sex-specific data for both sexes were part of the study evaluation phase of this systematic review. As shown in Supplemental Fig. 3, three of the studies (Lee et al., 2016; Maekawa et al., 2017; Monroy et al., 2008) included in the study evaluation are not considered further in the meta-analysis as they were considered *uninformative* largely due to critical study quality deficiencies across different domains (e.g., most often due to deficiencies in Participant Selection, Confounding, Analysis and Study Sensitivity domains). For example, in the Maekawa et al. (2017) study, critical deficiencies were identified due to lack of consideration of confounding and insufficient information provided on the sampling frame to evaluate potential for different biases.

The study characteristics, original study results, exposure distributions, and data re-expressions for the 27 studies included in the meta-analysis are shown in Table 1 and Table 2. For example, three studies only reporting sex-specific estimates for boys and girls (Lind et al., 2017; Robledo et al., 2015; Wang et al., 2016) (Table 2) were pooled based on inverse variance weights across sexes. We also rescaled six studies that used either log_10_ or log_2_ to natural logarithmic (ln) units (i.e., log_e_). We converted results that were presented in inter-quartile ranges or units of standard deviation changes into a ln-unit change. To increase the number of available studies, we also converted the three studies reporting results (Bach et al., 2016; Sagiv et al., 2018; Shoaff et al., 2018) in the natural scale (i.e., per ng/mL) into ln units (i.e., per ln (ng/mL)) using a percentile-based re-expression method.

**Fig. 1.**
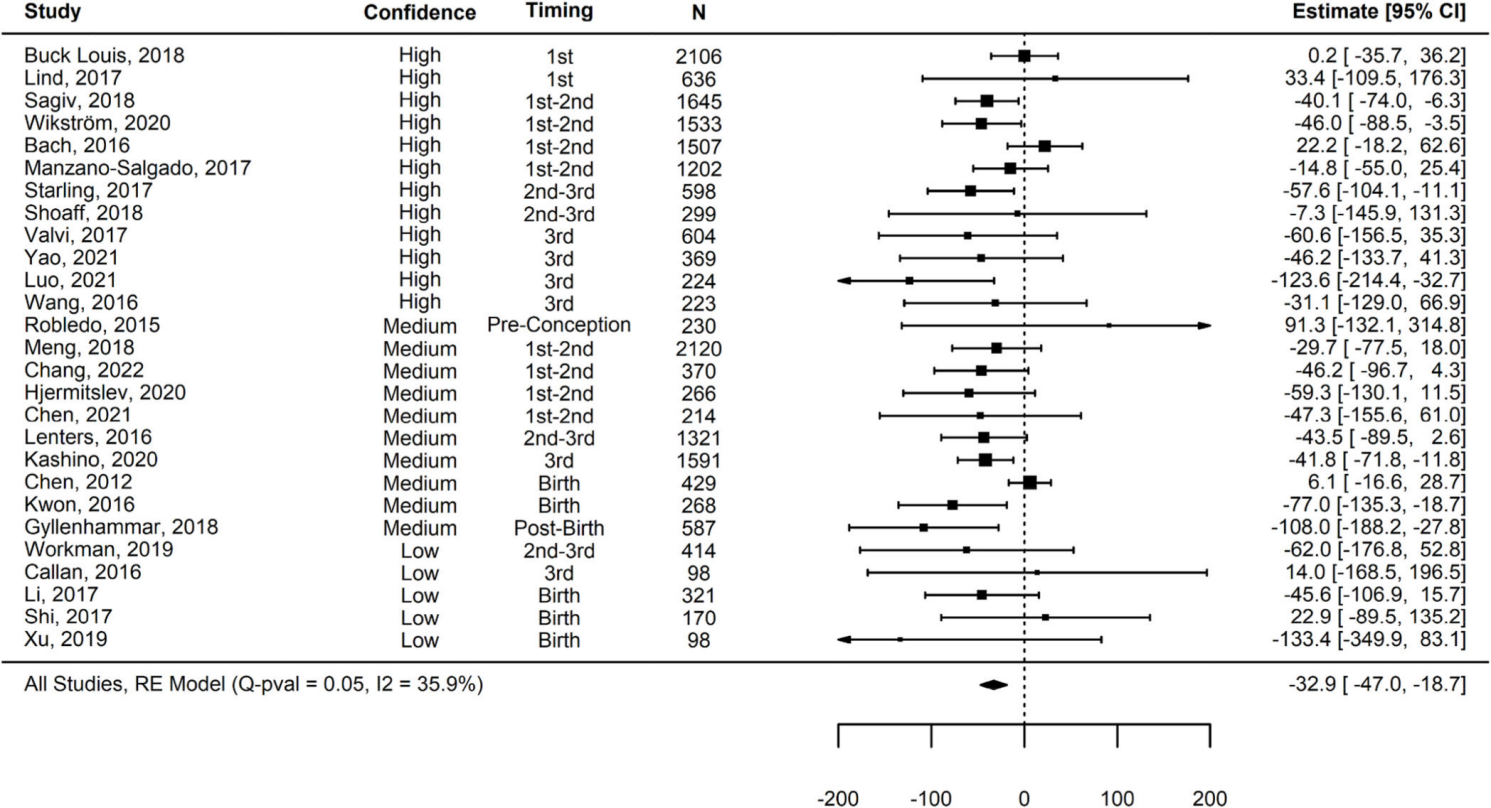
Forest plot of the 27 studies included for the meta-analysis on PFNA exposures and changes in birth weight. Arrows indicate where 95% CIs are truncated

**Fig. 2.**
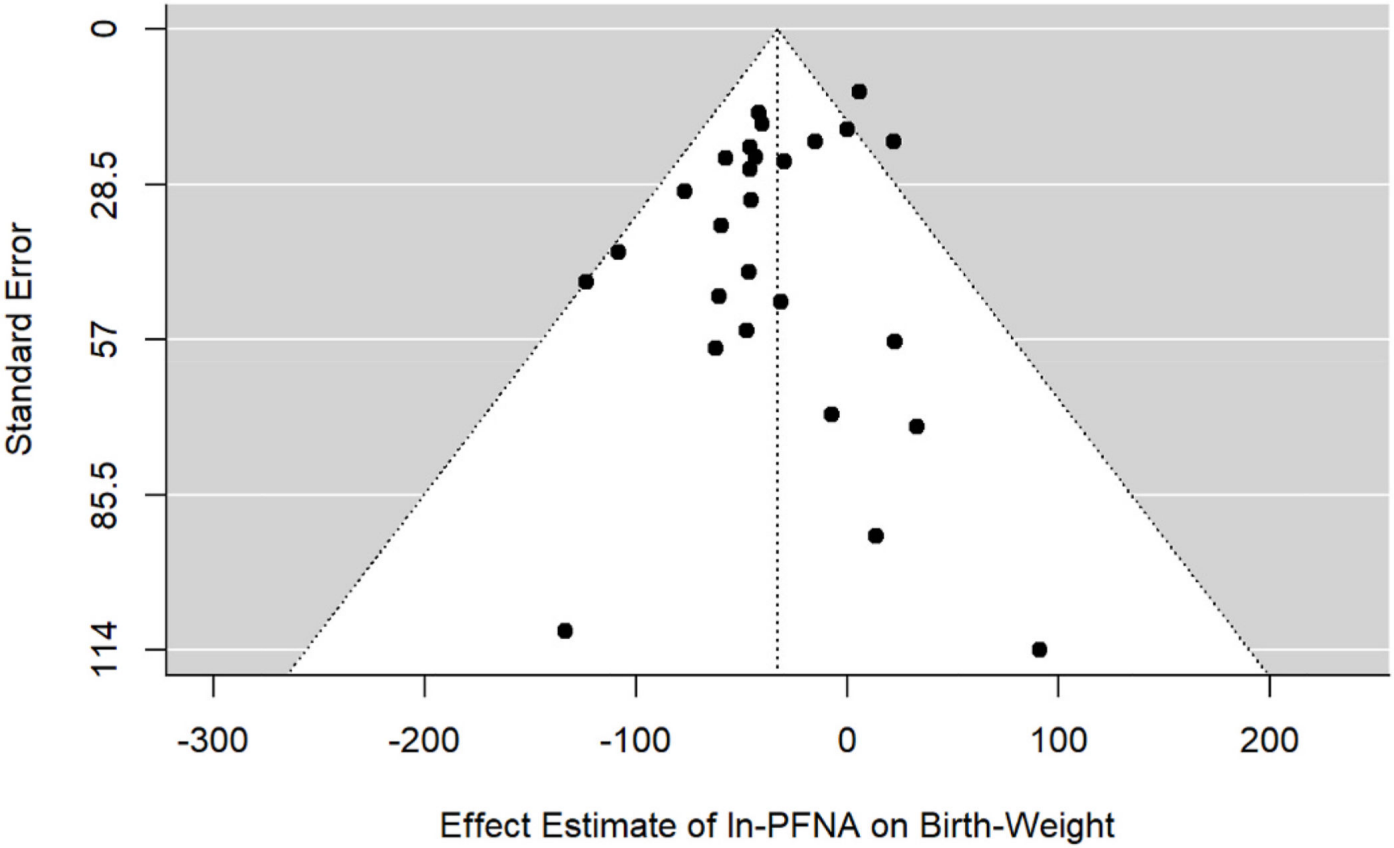
Funnel Plot of 27 studies examining PFNA exposures and birth weight differences.

Based on the meta-analysis of all 27 studies, we detected a 32.9 g (95% CI: −47, −18.7) decrease in birth weight per each ln ng/mL increase in PFNA (Fig. 1; Table 2). We did not detect evidence of between-study heterogeneity (p_Q_ = 0.05; I^2^ = 35.9%). The Egger’s regression test showed no significant relationship between the observed effect sizes and their standard error (p_E_ = 0.30) indicating no evidence of funnel plot asymmetry due to publication bias (Fig. 2).

### 3.1. Stratified meta-analysis - study confidence level

The 12 *high* confidence studies yielded a smaller pooled effect estimate (β = −28.0 g; 95% CI: −49.0, −6.9) than the 10 *medium* (β = −39.0 g; 95% CI: −61.8, −16.3) or five *low* (β = −36.9 g; 95% CI: −82.9, 9.1) confidence studies (Table 3); the differences between strata were not statistically significant (p = 0.77). The results for the limited number of *low* confidence studies (n = 5) were imprecise and showed no evidence of heterogeneity (I^2^ = 0%; p = 0.66). There was *low* heterogeneity amongst the *high* confidence studies (I^2^ = 38.8%; p = 0.11). There was *moderate* heterogeneity for the *medium* confidence studies (I^2^ = 48.1%; p = 0.03). Results similar in magnitude and precision to the overall pooled effect estimate were detected in the strata with *high* and *medium* confidence studies combined, since these constituted 22 of the 27 studies, (β = −32.9 g; 95% CI: −48.0, −17.8), with *moderate* heterogeneity detected (I^2^ = 42.2; p = 0.02).

### 3.2. Stratum-specific estimates - biomarker sample timing

The stratum-specific analysis based on 11 studies with sampling periods in earlier pregnancy (e.g., pre-conception, Trimester 1-only, and Trimester 1 and 2) yielded a smaller deficit (β = −22.0 g; 95% CI: 40.1, −4.0) compared to the ten mid- and late-pregnancy (β = −48.4 g; 95% CI: −67.7, −29.0) and six post-partum studies (β = −42.9 g; 95%CI: −88.0, 2.2) (Table 4). Mean BWT deficits increased over sample time, but there was overlap between the confidence bounds of the first two strata. Although we saw substantial differences across these strata and a gradient in deficits across timing, the hypothesis test for differences across any strata was not statistically significant (p = 0.14).

### 3.3. Leave-one-out analyses

We saw no evidence of *high* heterogeneity amongst any strata, including those with small sample sizes. For example, *moderate* between-study heterogeneity (I^2^ = 48%; p_Q_ = 0.03) was detected in the *medium* confidence group based on ten studies. Dropping the *medium* confidence Chen et al. (2012) study with umbilical cord samples (i.e., ‘at birth’ or post-pregnancy category) as part of the leave-one-out analysis led to the largest decrease in heterogeneity in the *medium* confidence sub-group (from 48% to 0%) and increased the mean BWT deficit from 39 g to 48 g per each ln-unit PFNA increase. Dropping this study from the post-pregnancy sampling strata changed the I^2^ value of 63% to 0% and beta coefficient from −43 g to −65 g. In contrast, the reduction in the I^2^ value from dropping Chen et al. (2012) in the complete set of 27 studies were more modest, reducing I^2^ from 36% to 21%. The overall summary estimate and that found for the *medium* and *high* confidence studies combined (−33 g for both) were robust to dropping the Chen et al. (2012) study (changed to −36.0 and 36.5 g, respectively, following study removal).

### 3.4. Continuous analysis of gestational age at sampling

To further examine the impact of sample timing with a continuous estimate of gestational age, we estimated the β coefficient representing the change in the effect of PFNA exposure on birth weight per each gestational week increase. Gestational age was measured as a combination of mean, median, range midpoints, weighted mean of means/medians/midpoint and exact week when samples were collected. Consistent with the binned sample timing findings, the β of −0.86 g (95% CI: −2.05, 0.32) indicated that as sampling time increased across studies, the pooled estimate of mean BWT deficits gets larger.

### 3.5. Sensitivity analysis

We saw few differences in the sample timing analyses comparing a three-strata to a binary two-strata approach (Table 4). Although differences across strata were not statistically significant in either case (p = 0.14 and 0.12 for the three- and two-strata approaches, respectively) compared to the early pregnancy or pre-conception (β = −22.0 g; 95% CI: −40.1, −4.0), both strategies for collapsing later sampled studies demonstrated results that were more than twice as large (10 mid-to late studies β = −48.4 g, 95% CI: −67.7, −29.0; 6 post studies β = −42.9 g, 95% CI: −88.0, 2.2; 16 late + post studies β = −44.5 g, 95% CI: −65.9, −23). Among the *high* confidence studies, there were six studies each that reported early biomarker sampling versus mid or late pregnancy sampling (no samples were taken post pregnancy among these high confidence studies). We ran a random effects model for the early and mid-/late studies within the *high* confidence strata and found that the pooled effect for the mid-/late *high* confidence studies was larger than the early *high* confidence studies by roughly four times (later samples β = −59.1 g, 95% CI: −91.0, −27.1; earlier samples β = −14.6, 95% CI: −38.4, 9.2). Among these *high* confidence studies, the early sampled studies exhibited moderate between-study heterogeneity (I^2^ = 44%), and differences between the estimates were also statistically significant (p-value = 0.03).

We compared the overall summary estimate for all 27 studies to the 24 studies originally reporting results based on any type of log transformation. The mean BWT deficit per unit change in PFNA for these 24 studies was slightly higher (β = −36.2 g; 95% CI: −51.2, −21.3) compared to the overall estimate of −32.9 g (95% CI: −47.0, −18.7). The three studies that reported results on the natural scale, requiring re-expression to the natural scale, were the *high* confidence publications (Bach et al., 2016; Sagiv et al., 2018; Shoaff et al., 2018). The sensitivity analysis of our assumption of a ln-unit transformation (β = −32.9 g) used in Kwon et al., 2016, in comparison to log_2_ (β = −33.0 g) and log_10_ (β = −30.6 g) did not change the overall pooled effect estimate reported here. Lastly, we carried out a sensitivity test to examine the appropriateness of choosing a random over a fixed effects framework and found evidence to support the choice of a random effects model (Supplemental Table 6).

## 4. Discussion

In this meta-analysis of PFNA and changes in BWT, we detected a 33 g decrease (95% CI: −47, −19) in mean BWT for each ln (ng/mL) PFNA exposure increase based on 27 studies. The large number of included studies allowed for a precise summary estimate and the ability to examine stratum specific estimates to explore potential sources of heterogeneity, including study confidence ratings. Our results were largely robust to various approaches, working assumptions and data expressions as all of the study confidence and timing stratum-specific deficits exceeded 22 g. We did not detect a pattern of BWT deficits based on overall study confidence descriptors, as the largest beta was seen for the *medium* confidence studies. The combined estimate for the 22 *medium* and *high* confidence studies (β = −33 g; 95% CI: −48, −18) was also similar in magnitude and precision to the overall pooled effect estimates based on all 27 studies. Our overall findings are consistent in magnitude to a recent publication by Gui et al. (2022) who reported a BWT deficit of −28 g (95%CI: −44.8, −11.8) per each increasing ln (ng/ml) PFNA exposure. That meta-analysis did not examine differences by sample timing and their analysis of continuous scaled results was based on considerably fewer studies (n = 14).

We evaluated the impact of sampling timing on summary estimates using both a discrete and continuous gestational age approach. Both analyses showed that sample timing was related to magnitude of associations that were reported. Grouping studies into a limited number of categories is challenging, since some studies were based on heterogeneous biomarker samples that spanned different time periods during or near pregnancy. This results in some overlap between group designations; for example, the “early” strata included studies that sampled from both trimesters 1 and 2, whereas studies that sampled in trimester 2 only are included in the “late” group. This may introduce dependence between the groups that our modeling approach does not account for. Nonetheless, our approach for the discrete variable analysis enabled an assignment to either an “early” or “late” pregnancy period. Earlier sampling results were attenuated relative to later sampling, but the early sampled strata still showed a sizeable BWT deficit (β = −22 g) with increasing PFNA exposures. The results stratified by sample timing were comparable irrespective of the binning approaches used. Our stratified sample timing meta-analysis and meta-regression findings are in contrast to earlier meta-analyses of PFOA data by Steenland et al. (2018) and PFOS by Dzierlenga et al., 2020 who both reported decrements in BWT only among studies with later sampling periods. One limitation in stratified analyses in previous studies and in our research is that, while the number of studies examined in the overall analysis is large (n = 27), some uncertainty exists for the stratified analyses with considerably fewer studies per strata. The pattern of attenuated results by sample timing was also seen in a sensitivity analysis of 12 *high* confidence studies, where we saw smaller magnitudes of BWT changes per each ln-unit PFNA increase in six early sampled studies (β = −15 g) compared to six later pregnancy sampled studies (β = −59 g).

We recognize that our meta-regression evaluating the impact of a continuous estimate of gestational age was targeted and limited in scope, and, thus, may be subject to some uncertainty. It is it well understood that measurement error in gestational age estimates based on either last menstrual period and/or ultrasound measures is anticipated to some degree. Another source of uncertainty is due to differences in the level of detail provided by studies to estimate the sampling timing gestational age measures. Similar to the approach used by Dzierlenga et al., 2020, we used a combination of measures of central tendency (median, mean, midpoint) to estimate gestational age at sample timing. This may serve as a limitation of the meta-regression, since most of the studies reported differing measures of centrality, necessitating the assumption that all measures of centrality are comparable. Furthermore, assuming a linear response in the continuous approach may be particularly influenced by measurement error associated with reported gestational age. We also anticipate some uncertainty in the classification of studies into discrete sample timing groupings due to measurement error in reported gestational age. However, the use of broad sample timing categories may in fact minimize the impact of misclassification and, therefore, may represent an advantage over the continuous approach. Our discrete variable results were internally consistent as they showed similar sample timing patterns irrespective of which categorical binning approaches were used. However, given these sources of measurement and variable reporting of these data in the literature, more research is needed to fully delineate the impact of PFAS sample timing on reported effects estimates.

A challenge of all meta-analyses is to maximize the sample size by increasing the comparability of published studies reporting disparate measures. To allow for more studies to be included in this meta-analysis and increase between-study comparability in presentation of results, we conducted various data re-expressions. For example, to allow for a more direct comparison, we rescaled results from studies that used log_2_ or log_10_ to ln. Our meta-analyses were performed using the β coefficient expressed per ln (ng/mL) of 27 studies, since the majority of the studies reported results on log scale. Similar to previous research (Dzierlenga et al., 2020), this analysis included re-expression of natural scale (i.e., untransformed) results to natural log scale. According to Linakis et al. (2021), data re-expression can result in bias that overestimates the truth by 20–25% in some situations. In our sensitivity analysis of 24 studies originally reported in log scale showed a comparable summary estimate (β = −36.2 g; 95% CI: −51.2, −21.3) to that based on all 27 studies (β = −32.9 g; 95% CI: −47.0, –18.7) which included three studies re-expressed on log scale (Bach et al., 2016; Sagiv et al., 2018, Shoaff et al., 2018). These data showed only a minor difference (~10%) and suggest that our data re-scaling and re-expressions had minimal impact on the overall summary estimate. This indicates that any bias from re-expressions used in the dataset here was likely small and that the summary estimate of 27 studies may better reflect the overall weight of evidence as it provides a more comprehensive evaluation of the literature with increased precision.

Another study strength was the detailed evaluation of study sensitivity, risk of bias domains and overall study confidence which allowed for examination of different strata. Some of the primary factors that led to lower confidence scores primarily included deficiencies in Participant Selection, Confounding or Analysis domains. Some sources of uncertainties related to confounding included the unknown impact of pregnancy hemodynamics as noted before and the potential impact of other co-occurring PFAS exposures. While there are many ways to examine exposures to environmental mixtures, including interactions or summed mass of all constituent species, most studies explore exposure to single PFAS in separate models. This is likely related to the fact that many current policy actions are focused on single constituents, although this is beginning to change. Mechanisms of action may be similar or different for chemicals within any PFAS mixture, and knowledge of these interactions is currently limited but may be important as noted in recent publications (Anderson et al., 2022; Ojo et al., 2021; Rosato et al., 2022; Zhuang et al., 2021). In addition to some uncertainty related to potential interactions across PFAS, there is potential for confounding of associations with health effects across PFAS. Some of the main challenges related to statistical adjustment of correlated PFAS include concerns over collinearity and co-amplification bias (Weisskopf et al., 2018). Nonetheless, studies were downgraded in the Confounding domain, for example, if they did not consider multiple PFAS exposures in their regression analyses or were unable to rule out confounding through other study design features. For comparability across studies, our meta-analysis includes PFNA only regression models (i.e., single PFAS models) when other multi-PFAS model results were provided. Our assessment of single- and multi-PFAS model differences found that all three (Starling et al., 2017; Lenters et al., 2016; Manzano-Salgado et al., 2017) studies with multipollutant models examining mean BWT differences reported larger PFNA associations upon adjustment of additional PFAS (range: 20 to −92 g per each ln (ng/mL) increase). Only one of two studies (Starling et al., 2017; Lenters et al., 2016) identified PFNA as an important contributor in elastic net models albeit with smaller deficits reported in Starling (β = −33 g per each ln (ng/mL) increase).

The study confidence stratified analysis also allowed for consideration of other factors that impact between-study heterogeneity beyond sample timing. Overall, we detected “low” between-study heterogeneity across all 27 studies and “moderate” heterogeneity among the *medium* confidence studies. One source of uncertainty in the *medium* confidence strata came from Kwon et al. (2016), which did not specify the log base of the reported beta coefficients. We assumed a natural log scale was used and confirmed the robustness of this assumption on the pooled estimate based on sensitivity tests to other commonly used log scales, log_2_ and log_10_ (Supplemental Table 4). Although the *medium* confidence group had a moderate I^2^ statistic (48%), our leave-one-out analysis showed that the stratum-specific results were robust (similar β and I^2^) to the exclusion of the Kwon et al. (2016) study. Given the heterogeneity detected for the *medium* confidence group, there was more variability in stratum-specific estimates related to this subset of studies. The leave-one-out analyses identified Chen et al. (2012) as a primary contributor to this heterogeneity presumably due to the precise null result originally reported in their study. Although this study had minimal impact on the overall study estimate based on all 27 studies, the between study-heterogeneity statistics were much smaller when it was not included in the smaller sub-strata.

A limitation of our meta-analysis is that we did quantitatively differentiate or estimate the amount of bias or variation anticipated when studies measured different biomarkers such as umbilical cord versus maternal serum, plasma, and whole blood. For example, according to a physiologically-based pharmacokinetic model by Verner et al. (2015), the use of cord serum or plasma would overestimate the beta coefficent by −1.26 g/ng/ml compared with maternal serum sampled at 40 weeks of gestation. As noted previously, we saw larger effects amongst the later sampled studies which included the umbilical cord studies. We did not attempt to further isolate results for this sample type sub-group to compare, given that there may be other sources of between-study heterogeneity that would need to be considered to fully delineate any differences. Previous PFAS meta-analysis and our research here on PFNA that demonstrate potential sources of error based on sample type and timing hopefully will encourage more data analysis and reporting standardization efforts (e.g. biomarker sample timing detail) to increase comparability across studies in systematic reviews.

These findings are novel especially in comparison to what has been described for other PFAS such as PFOS and PFOA. This may have important ramifications for risk assessment and management decision-making which has been complicated by the myriad PFAS in the environment as legacy long-chained PFAS are being replaced with short-chain PFAS. There is some recent evidence that PFNA levels, unlike PFOS, PFOA and PFHxS, are decreasing in the U.S. but are increasing in several regions, including East Asia and Europe (Fan et al., 2022). As noted above, more research on which PFAS constituents to best target in combination for toxicological and epidemiological studies is needed. Limited information also exists regarding potential mechanisms in epidemiological studies of fetal growth restriction, but include epigenetic changes, maternal circulating nutrients (e.g., glucose), as well as reproductive, adinopekine, and thyroid hormones. Although some epidemiological studies have recently examined different covariates as possible mediators or modifiers, the biological mechanisms of fetal growth restriction related to PFNA are not clearly understood. For example, studies have shown that thyroid hormones (no evidence) and maternal glucose (explained only 2.5% of the total effect) were either very weak or not identified as mediators (Xiao et al., 2020; Starling et al., 2017). Gestational diabetes was also shown to not be either a modifier or mediator reported associations between BWT and PFNA exposures (Valvi et al., 2017). These studies represent important information even if considerable uncertainty remains on potential mechanistic pathways.

## 5. Conclusion

Overall, we detected a sizeable deficit in mean BWT per each ln increase of PFNA that was robust across numerous analyses and showed little evidence of either publication bias or between-study heterogeneity. Our approach leverages data re-expression techniques that allow a greater number of studies to be included and facilitate better evaluation of between-study heterogeneity. In contrast to earlier studies on PFOA and PFOS which dichotomized their studies into those that sampled PFAS either early or late in pregnancy, our analysis showed deficits across all sampling periods irrespective of whether post-partum samples were differentiated from late pregnancy samples. So, although we observed attenuated effect estimates in earlier periods, all stratified study confidence and timing estimates were at least 22 g per each ln-unit increase in PFNA exposure. In contrast to previous PFOA and PFOS meta-analyses, our results showed adverse associations across all sampling periods despite some attenuation of the earlier periods. Importantly, this is the first PFAS meta-analysis to report BWT deficits that do not appear to be fully explained by sample timing considerations and pregnancy hemodynamics.

In conclusion, the weight of evidence for mean BWT deficits here are stronger for PFNA than have been reported for other PFAS. Although considerable uncertainty remains regarding the complex patterns on influence from pregnancy hemodynamics, our meta-regression and stratified analyses of biomarkers based on earlier pregnancy samples may better reflect a true association if later samples are anticipated to be a source of bias in studies. Future meta-analyses should further examine the potential impacts of PFNA on developmental effects especially as these types of systematic review efforts can be instrumental to support regulatory decision-making (Gonzales-Barron and Butler, 2011; Goodman et al., 2010; Martin et al., 2018).

## Supplementary Material

Supplement1

Supplementary data to this article can be found online at https://doi.org/10.1016/j.envres.2023.115357.

## Figures and Tables

**Table 1:** Characteristics of 27 studies with continuous PFNA exposures included in the meta-analysis.

Reference	Study name and years	Country	Study design	n	Study confidence	Matrix	Exposure window	Adjustment factors

Bach et al., 2016	Aarhus Birth Cohort (2008–2013)	Denmark	Cohort	1,507	High	Maternal serum	T 1–2	education, maternal age, maternal pre-pregnancy BMI
Buck Louis et al., 2018	NICHD Fetal Growth Studies (2009–2013)	United States	Cohort	2,106	High	Maternal plasma	T 1	age, education, pre-pregnancy BMI, infant sex, serum cotinine
Callan et al., 2016	AMETS (2008–2011)	Australia	Cross-sectional	98	Low	Maternal blood	T 3	gestational age, infant sex, maternal BMI, maternal height, maternal weight gain during pregnancy
Chang et al., 2022	Emory University African American Vaginal, Oral, and Gut Microbiome in Pregnancy Study (2014–2018)	United States	Cohort	370	Medium	Maternal serum	T 1–2	maternal age, education, parity, body mass index, tobacco use, marijuana use, infant sex
Chen et al., 2021	Shanghai Birth Cohort (2013–2015)	China	Cohort	214	Medium	Maternal plasma	T 1–2	maternal age, prepregnancy BMI, educational level, occupation, family income, smoking and alcohol use, parity, fetal sex
Chen et al., 2012	Taiwan Birth Panel Study (2004–2005)	Taiwan	Cross-sectional	429	Medium	Cord blood plasma	Birth	delivery type, education, gestational age, head circumference, infant sex, ln-transformed cord blood cotinine levels, maternal age, maternal pre-pregnancy BMI, neonatal birth length, parity
Gyllenhammar et al., 2018	POPUP (1996–2011)	Sweden	Cross-sectional	381	Medium	Maternal serum	3 weeks post-birth	fish intake, maternal age, maternal pre-pregnancy BMI maternal weight gain during pregnancy, maternal weight loss after delivery, sampling year, smoking, years of education
Hjermitslev et al., 2020	ACCEPT (2013–2015)	Greenland	Cohort	270	Medium	Maternal Serum	T 1–3	maternal age, pre-pregnancy BMI, parity, cotinine, alcohol
Kashino et al., 2020	Hokkaido Study on Environment and Children’s Health (2003 and 2009)	Japan	Cohort	1,985	Medium	Maternal serum	T 3	maternal age at delivery, pre-pregnancy maternal BMI, maternal education, plasma cotinine concentration during pregnancy, parity, sex of the infant, gestational age
Kwon et al., 2016	EBGRC (2006–2010)	South Korea	Cross-sectional	268	Medium	Cord blood serum	Birth	alcohol consumption, gender, gestational age, maternal age, maternal pre-pregnancy body mass index, parity
Lenters et al., 2016	INUENDO (2002–2004)	Ukraine	Cohort	1,321	Medium	Maternal serum	T 2–3	alcohol consumption, gestational age, infant sex, maternal age, maternal height, maternal pre-pregnancy BMI, parity, serum cotinine, study population, vitamin D
Li et al., 2017	Guangzhou Birth Cohort (2013)	China	Cohort	321	Low	Cord blood serum	Birth	gestational age, delivery, education, parity, infant sex, maternal age, pregnancy-induced hypertension, gestational diabetes, anemia
Lind et al., 2017	Odense Child Cohort (2010–2012)	Denmark	Cohort	638	High	Maternal serum	T 1	gestational age, maternal pre-pregnancy BMI, parity, smoking
Luo et al., 2021	Guangzhou (2017–2019)	China	Cohort	224	High	Maternal plasma	T 3	maternal age, pre-pregnancy body mass index, education, parity, environmental tobacco smoke exposure, alcohol drinking, gestational age, newborn sex
Manzano-Salgado et al., 2017	INMA cohort (2003–2008)	Spain	Cohort	1,202	High	Maternal plasma	T 1	fish intake, GFR, maternal age, maternal pre-pregnancy BMI, parity
Meng et al., 2018	Danish National Birth Cohort (1996–2002)	Denmark	Cohort	3,535	Medium	Maternal plasma	T 1–2, at birth	alcohol consumption, gestational age at blood draw, infant birth year, infant sex, maternal age, maternal pre-pregnancy BMI, parity, smoking during pregnancy, socio-occupational status
Robledo et al., 2015	LIFE Study (2005–2009)	United States	Cohort	234	Medium	Maternal serum	Pre-conception	difference in parental age, individual and partner sum of remaining chemical concentrations in each chemical’s respective class, infant gender, maternal age, maternal pre-pregnancy BMI, maternal serum lipids, paternal serum lipids, serum cotinine
Sagiv et al., 2018	Project Viva (1999–2002)	United States	Cohort	1,645	High	Maternal serum	T 1–2	education, gestational age at blood draw, history of breastfeeding, household income, maternal age, maternal pre-pregnancy BMI, newborn sex, parity, paternal education, race/ethnicity, smoking during pregnancy
Shi et al., 2017	Haidan Hospital (2012)	China	Cross-sectional	170	Low	Cord blood serum	Birth	gestational age, infant gender, maternal age, maternal pre-pregnancy BMI, parity
Shoaff et al., 2018	HOME (2003–2006)	United States	Cohort	345	High	Maternal serum	T 2–3	prenatal vitamin use, fruit/vegetable and fish consumption during pregnancy, food security, mid-pregnancy BMI, depressive symptoms, serum cotinine, parity, education, income, insurance, marital status, race, maternal age at delivery
Starling et al., 2017	Healthy Start cohort (2009–2014)	United States	Cohort	628	Medium	Maternal serum	T 2–3	BMI, education, gestational age at birth, gestational age at blood draw, gestational weight gain, gravidity, infant sex, maternal age, race/ethnicity, smoking during pregnancy
Valvi et al., 2017	Faroe Islands (1997–2000)	Denmark	Cohort	604	High	Maternal serum	T 3	education, infant sex, maternal age at delivery, maternal BMI, parity, smoking during pregnancy
Wang et al., 2016	Taiwan Maternal and Infant Cohort Study (2000–2001)	Taiwan	Cohort	223	High	Maternal serum	T 3	family annual income, maternal age at delivery, maternal education, maternal pre-pregnancy BMI, maternal previous live births
Wikström et al., 2020	SELMA (2007–2010)	Sweden	Cohort	1,533	High	Maternal serum	T 1–2	sex, gestational age, parity, maternal weight, and cotinine
Workman et al., 2019	Canadian Healthy Infant Longitudinal Development Study (2010–2011)	Canada	Cohort	414	Low	Maternal plasma	T 2–3	maternal age, maternal smoking during pregnancy, maternal high blood pressure during pregnancy, maternal diabetes during pregnancy, parity, infant sex
Xu et al., 2019	Women’s Hospital of the School of Medicine (2016–2017)	China	Cross-sectional	98	Low	Cord blood serum	Birth	previous abortions, alcohol consumption, birth gender, education, gestational age, job, maternal age, maternal BMI, parity, pregnancy weight gain
Yao et al., 2021	Laizhou Wan Birth Cohort (2010–2013)	China	Cohort	369	High	Maternal serum	T 3	maternal age at delivery, pre-pregnancy BMI, gestational weight gain, ethnic groups, maternal education, passive smoking during pregnancy, parity

Abbreviations: T 1: Trimester 1; T 2: Trimester 2; T 3: Trimester 3; BMI: Body Mass Index; GFR: Glomerular Filtration Rate; Cohort: Birth cohort study with longitudinal follow-up to and/or beyond birth (e.g., a Prospective Cohort).

**Table 2:** Original study and re-expressed results based on reported PFNA exposure distributions.

Study	Exposure Summary Mean/q50/GM (Dispersion)	Samples BLOD	Exposure Distribution in ng/ml (μ, σ)	Original β β (LCL, UCL)	Unit	Re-expressed β (g/ln(ng/ml)) β (LCL, UCL)
Bach et al., 2016	0.80 (0.60–1.00 IQR ng/ml)	1%	(−0.22, 0.38)	11.0 (−9.0, 31.0)	g/IQR (ng/ml)	22.2 (−18.2, 62.6)
Buck Louis et al., 2018	0.76 (0.53–1.16 IQR ng/ml)	0	(−0.27, 0.58)	0.1 (−20.9, 21.2)	g/SD ln(1 + ng/ml)	0.2 (−35.7, 36.2)
Callan et al., 2016	0.30 (0.18 SD ng/ml)	0	(−1.36, 0.55)	14.0 (−169.0, 196.0)	g/ln(ng/ml)	14.0 (−169.0, 196.0)
Chang et al., 2022	0.24 (2.30 GSD ng/ml)	3%	(−1.43, 0.83)	−32.0 (−67.0, 3.0)	g/log2(ng/ml)	−46.2 (−96.7, 4.3)
Chen et al., 2012	2.36 (4.74 GSD ng/ml)	32%	(0.86, 1.56)	6.1 (−16.6, 28.7)	g/ln(ng/ml)	6.1 (−16.6, 28.7)
Chen et al., 2021	2.33 (1.61–3.35 IQR ng/ml)	0	(0.85, 0.54)	−47.3 (−155.6, 61.0)	g/ln(ng/ml)	−47.3 (−155.6, 61.0)
Gyllenhammar et al., 2018	0.41 (0.31–0.57 IQR ng/ml)	0	(−0.89, 0.45)	−108.0 (−188.2, −27.8)	g/ln(ng/ml)	−108.0 (−188.2, −27.8)
Hjermitslev et al., 2020	1.15 (0.85–1.59 IQR ng/ml)	0	(0.14, 0.47)	−59.3 (−130.0, 11.6)	g/ln(ng/ml)	−59.3 (−130.0, 11.6)
Kashino et al., 2020	1.20 (0.90–1.60 IQR ng/ml)	0.1%	(0.18, 0.43)	−96.2 (−165.3, −27.1)	g/log10(ng/ml)	−41.8 (−71.8, −11.8)
Kwon et al., 2016	0.16 (2.14 GSD ng/ml)	NR	(−1.83, 0.76)	−77.0 (−135.3, −18.7)	g/ln(ng/ml)	−77.0 (−135.3, −18.7)
Lenters et al., 2016	0.65 (1.03 2SD ln(ng/ml))	0	(−0.43, 0.51)	−44.7 (−92.0, 2.7)	g/2SD ln(ng/ml)	−43.5 (−89.5, 2.6)
Li et al., 2017	0.23 (0.29 SD ng/ml)	22%	(−1.95, 0.98)	−45.6 (−106.9, 15.8)	g/ln(ng/ml)	−45.6 (−106.9, 15.8)
Lind et al., 2017 Male	0.70 (0.50–0.90 IQR ng/ml)	NR	(−0.36, 0.44)	−36.0 (−140.0, 69.0)	g/ln(ng/ml)	−36.0 (−140.0, 69.0)
Lind et al., 2017 Female	0.70 (0.50–0.90 IQR ng/ml)	NR	(−0.36, 0.44)	110.0 (−13.0, 232.0)	g/ln(ng/ml)	110.0 (−13.0, 232.0)
Luo et al., 2021	0.50 (0.39–0.67 IQR ng/ml)	0	(−0.69, 0.40)	−123.6 (−214.4, −32.7)	g/ln(ng/ml)	−123.6 (−214.4, −32.7)
Manzano-Salgado et al., 2017	0.66 (0.36 SD ng/ml)	0	(−0.55, 0.51)	−10.3 (−38.1, 17.6)	g/log2(ng/ml)	−14.8 (−55.0, 25.4)
Meng et al., 2018	0.50 (0.40–0.60 IQR ng/ml)	8%	(−0.69, 0.30)	−20.6 (−53.7, 12.5)	g/log2(ng/ml)	−29.7 (−77.5, 18.0)
Robledo et al., 2015 Male	1.57 (0.32 SD ln(ng/ml))	NR	(0.45, 0.32)	62.7 (−32.1, 157.4)	g/SD ln(ng/ml)	196.6 (−100.6, 493.8)
Robledo et al., 2015 Female	1.21 (0.31 SD ln(ng/ml))	NR	(0.19, 0.31)	−10.1 (−111.5, 91.3)	g/SD ln(ng/ml)	−32.1 (−355.1, 290.8)
Sagiv et al., 2018	0.70 (0.50–1.00 IQR ng/ml)	NR	(−0.36, 0.51)	−28.2 (−52.0, −4.4)	g/IQR (ng/ml)	−40.1 (−74.0, −6.3)
Shi et al., 2017	0.22 (0.12 SD ng/ml)	0	(−1.63, 0.51)	52.7 (−206.0, 311.4)	g/log10(ng/ml)	22.9 (−89.5, 135.2)
Shoaff et al., 2018	0.90 (0.70–1.10 33–67 IQR ng/ml)	NR	(−0.11, 0.51)	−8.0 (−159.5, 143.5)	g/ng/ml	−7.3 (−145.9, 131.2)
Starling et al., 2017	0.40 (0.30–0.60 IQR ng/ml)	1%	(−0.92, 0.51)	−57.6 (−104.1, −11.2)	g/ln(ng/ml)	−57.6 (−104.1, −11.2)
Valvi et al., 2017	0.59 (0.46–0.79 IQR ng/ml)	0	(−0.53, 0.40)	−42.0 (−108.0, 25.0)	g/log2(ng/ml)	−60.6 (−155.8, 36.1)
Wang et al., 2016 Male	1.55 (0.92–2.60 IQR ng/ml)	3%	(0.44, 0.77)	20.0 (−60.0, 110.0)	g/ln(ng/ml)	20.0 (−60.0, 110.0)
Wang et al., 2016 Female	1.58 (0.84–2.42 IQR ng/ml)	3%	(0.46, 0.78)	−80.0 (−160.0, 0.0)	g/ln(ng/ml)	−80.0 (−160.0, 0.0)
Wikström et al., 2020	0.53 (0.39–0.73 IQR ng/ml)	0	(−0.63, 0.46)	−46.0 (−89.0, −4.0)	g/ln(ng/ml)	−46.0 (−89.0, −4.0)
Workman et al., 2019	0.44 (0.31 SD ng/ml)	4%	(−1.02, 0.63)	−62.0 (−176.8, 52.8)	g/ln(ng/ml)	−62.0 (−176.8, 52.8)
Xu et al., 2019	0.28 (0.14 SD ng/ml)	0	(−1.39, 0.47)	−307.2 (−805.7, 191.2)	g/log10(ng/ml)	−133.4 (−349.9, 83)
Yao et al., 2021	0.81 (0.57–1.07 IQR ng/ml)	0	(−0.21, 0.47)	−46.2 (−133.7, 41.3)	g/ln(ng/ml)	−46.2 (−133.7, 41.3)

Abbreviations: Q25: 25% percentile; Q50: 50% percentile (median); Q75; 75% percentile; BLOD: below limit of detection; SD: standard deviation; GSD geometric standard deviation; SE: standard error; β: beta coefficient; LCI: lower 95% confidence interval; UCI: upper 95% confidence interval; IQR: inter-quartile range; NR: not reported.

**Table 3:** Random effect estimates (β are in g/ln(ng/ml) of mean birth weight differences (and tests for heterogeneity) for PFNA exposures from 27 studies stratified by study confidence.

Set of Studies	*n*	β (g per ln (ng/mL))	95% Confidence Interval	I^2^ (%)	p_Q_

All Studies	27	−32.9	−47.0, −18.7	35.9	0.05
High Confidence	12	−28.0	−49.0, −6.9	38.8	0.11
Medium Confidence	10	−39.0	−61.8, −16.3	48.1	0.03
Low Confidence	5	−36.9	−82.9, 9.1	0.0	0.66
High + Medium Confidence	22	−32.9	−48.0, −17.8	42.2	0.02

Symbols and abbreviations: n = sample size; β = combined estimate of change in birth weight (g) per ln (ng/mL) PFNA exposure; I^2^ = % variation in the pooled effect due to study heterogeneity; p_Q_ = p-value for Cochrane’s Q test for heterogeneity.

**Table 4:** Random effect estimates (β are in g/ln(ng/ml) of mean birth weight differences (and tests for heterogeneity) for PFNA exposures from 27 studies stratified by sample timing.

Set of Studies	*n*	β (g per ln (ng/mL))	95% Confidence Interval	I^2^ (%)	p_Q_

All Studies	27	− 32.9	− 47.0, − 18.7	35.9	0.05
Early Pregnancy^a^	11	− 22.0	− 40.1, − 4.0	25.9	0.26
Mid and Late-Pregnancy	10	− 48.4	− 67.7, − 29.0	0.0	0.91
Post-Pregnancy	6	− 42.9	− 88.0, 2.2	63.1	0.01
Mid and Late + Post Pregnancy	16	− 44.5	− 65.9, − 23	40.0	0.05

Symbols and abbreviations: n = sample size; β = combined estimate of change in birth weight (g) per ln (ng/mL) PFNA exposure; I^2^ = % variation in the pooled effect due to study heterogeneity; p_Q_ = p-value for the Cochrane’s Q test for heterogeneity.

a Early Pregnancy or Pre-conception.

## Data Availability

Data will be made available on request.
