# Supplementary material for: Systematic review and meta-analysis of birth weight and PFNA exposures
Source: Environ Res. Author manuscript; Available in PMC 2025 Jun 11. (PMC12153499; doi:10.1016/j.envres.2023.115357)
Supplement: Supplement1 [file NIHMS2082567-supplement-Supplement1.docx]

*Supplemental Files*

**Supplemental Table 1. Populations, Exposures, Comparators, Outcome Criteria (PECO)*.**

| PECO element | Evidence |
| --- | --- |
| Populations | Human: Any population and life stage (occupational or general population, including children and other sensitive populations). The following study designs will be included: controlled exposure, cohort, case control, and cross-sectional. (Note: Case reports and case series will be tracked as potential supplemental material.)  Animal: Nonhuman mammalian animal species (whole organism) of any life stage (including preconception, in utero, lactation, peripubertal, and adult stages).  Other: In vitro, in silico, or nonmammalian models of genotoxicity. (Note: Other in vitro, in silico, or nonmammalian models will be tracked as potential supplemental material.) |
| Exposures | Human: Studies providing quantitative estimates of PFNA exposure based on administered dose or concentration, biomonitoring data (e.g., urine, blood, or other specimens), environmental or occupational setting measures (e.g., water levels or air concentrations, residential location and/or duration, job title, or work title). (Note: Studies that provide qualitative, but not quantitative, estimates of exposure will be tracked as supplemental material.)  Animal: Oral or inhalation studies including quantified exposure to PFNA based on administered dose, dietary level, or concentration. (Note: Non-oral and non-inhalation studies will be tracked as potential supplemental material.) PFNA mixture studies are included if they employ an experimental arm that involves exposure to a single PFNA. (Note: Other PFNA mixture studies are tracked as potential supplemental material.)  Studies must address exposure to following: PFNA (CASRN 375-95-1), PFNA sodium salt (CASRN 21049-39-8), or PFNA ammonium salt (CASRN 4149-60-4). |
| Comparators | Human: A comparison or reference population exposed to lower levels (or no exposure/exposure below detection levels) or for shorter periods of time.  Animal: Includes comparisons to historical controls or a concurrent control group that is unexposed, exposed to vehicle-only or air-only exposures. [Note: experiments including exposure to PFAS across different durations or exposure levels without including one of the aforementioned control groups will be tracked as potential supplemental material (e.g., for evaluating key science issues; Section 2.4 of the protocol)]. |
| Outcomes | All cancer and noncancer health outcomes. (Note: Other than genotoxicity studies, studies including only molecular endpoints [e.g., gene or protein changes; receptor binding or activation] or other non-phenotypic endpoints addressing the potential biological or chemical progression of events contributing towards toxic effects will be tracked as potential supplemental material [e.g., for evaluating key science issues; Section 2.4 of the protocol].) |
| PBPK models | Studies describing physiologically based pharmacokinetic (PBPK) and other pharmacokinetic (PK) models for PFNA (CASRN 375-95-1), PFNA sodium salt (CASRN 21049-39-8), or PFNA ammonium salt (CASRN 4149-60-4). |

*PECO criteria here is derived from the parent IRIS PFAS PECO based on several PFAS (US EPA, 2021).

**Supplemental Table 2. Literature Search Terms.**

| **Search** | **Search Strategy** |
| --- | --- |
| **Pub Med** | |
| Search Terms | "375-95-1"[rn] OR "2,2,3,3,4,4,5,5,6,6,7,7,8,8,9,9,9-heptadecafluorononanoic acid"[tw] OR "Nonanoic acid, 2,2,3,3,4,4,5,5,6,6,7,7,8,8,9,9,9-heptadecafluoro-"[tw] OR "Nonanoic acid, heptadecafluoro-"[tw] OR "Perfluoro-n-nonanoic acid"[tw] OR "Perfluorononan-1-oic acid"[tw] OR "Perfluorononanoate"[tw] OR "Perfluorononanoic acid"[tw] OR "Perfluorononanonic acid"[tw] OR "Perfluoropelargonic acid"[tw] OR "heptadecafluorononanoic acid"[tw] OR (("PFNA"[tw] OR "C 1800"[tw]) AND (fluorocarbon*[tw] OR fluorotelomer*[tw] OR polyfluoro*[tw] OR perfluoro-*[tw] OR perfluoroa*[tw] OR perfluorob*[tw] OR  perfluoroc*[tw] OR perfluorod*[tw] OR perfluoroe*[tw] OR perfluoroh*[tw] OR perfluoron*[tw] OR perfluoroo*[tw] OR perfluorop*[tw] OR perfluoros*[tw] OR perfluorou*[tw] OR perfluorinated[tw] OR fluorinated[tw] OR PFAS[tw] OR PFOS[tw] OR PFOA[tw])) |
| Literature Update and Additional PFNA Synonyms Search Terms | ((("2,2,3,3,4,4,5,5,6,6,7,7,8,8,9,9,9-heptadecafluorononanoic acid" [tw] OR "Nonanoic acid, 2,2,3,3,4,4,5,5,6,6,7,7,8,8,9,9,9-heptadecafluoro-" [tw] OR "Nonanoic acid, heptadecafluoro-" [tw] OR "Perfluoro-n-nonanoic acid" [tw] OR "Perfluorononan-1-oic acid" [tw] OR "Perfluorononanoate" [tw] OR "Perfluorononanoic acid" [tw] OR "Perfluorononanonic acid" [tw] OR "Perfluoropelargonic acid" [tw] OR "heptadecafluorononanoic acid" [tw] OR "PFNA" [tw] OR "C 1800" [tw] OR "Methyl-n1-Perfluorononanoic acid" [tw] OR "PFNA-n1CH3" [tw] OR "EINECS 206-801-3" [tw] OR "Heptadecafluornonansaeure" [tw] OR "Heptadekafluornonansaeure" [tw] OR "Perfluornonansaeure" [tw] OR "Perfluorononanoic acid (PFNA)" [tw] OR "UNII-5830Z6S63M" [tw] OR "perfluoro-n-nonanoic acid" [tw] OR "perfluorononan-1-oic acid" [tw] OR "perfluorononanoic acid" [tw] OR "Ammonium Perfluorononanoate" [tw] OR "Ammonium perfluorononanoate" [tw] OR "PFNA-H3N" [tw]))) AND ("2017/01/01"[Date - Publication] : "3000"[Date - Publication]) |
| **Web of Science** | |
| Search Terms | ((TS=PFNA OR TS="C 1800") AND TS=(fluorocarbon* OR fluorotelomer* OR polyfluoro* OR perfluoro-* OR perfluoroa* OR perfluorob* OR perfluoroc* OR perfluorod* OR perfluoroe* OR perfluoroh* OR perfluoron* OR perfluoroo* OR perfluorop* OR perfluoros* OR perfluorou* OR perfluorinated OR fluorinated OR PFAS OR PFOS OR PFOA)) OR TS="2,2,3,3,4,4,5,5,6,6,7,7,8,8,9,9,9-heptadecafluorononanoic acid" OR TS="Nonanoic acid, 2,2,3,3,4,4,5,5,6,6,7,7,8,8,9,9,9-heptadecafluoro-" OR TS="Nonanoic acid, heptadecafluoro-" OR TS="Perfluoro-n-nonanoic acid" OR TS="Perfluorononan-1-oic acid" OR TS="Perfluorononanoate" OR TS="Perfluorononanoic acid" OR TS="Perfluorononanonic acid" OR TS="Perfluoropelargonic acid" OR TS="heptadecafluorononanoic acid” |
| Literature Update and Additional PFNA Synonyms | (TS="PFNA" OR TS="C 1800" OR TS="2,2,3,3,4,4,5,5,6,6,7,7,8,8,9,9,9-heptadecafluorononanoic acid" OR TS="Nonanoic acid, 2,2,3,3,4,4,5,5,6,6,7,7,8,8,9,9,9-heptadecafluoro-" OR TS="Nonanoic acid, heptadecafluoro-" OR TS="Methyl-n1-Perfluorononanoic acid" OR TS="PFNA-n1CH3" OR TS="EINECS 206-801-3" OR TS="Heptadecafluornonansaeure" OR TS="Heptadekafluornonansaeure" OR TS="Perfluornonansaeure" OR TS="Perfluorononanoic acid (PFNA)" OR TS="UNII-5830Z6S63M" OR TS="perfluoro-n-nonanoic acid" OR TS="perfluorononan-1-oic acid" OR TS="perfluorononanoic acid" OR TS="Ammonium Perfluorononanoate" OR TS="Ammonium perfluorononanoate" OR TS="PFNA-H3N") AND PY=2017-2022 |
| **Toxline** | |
| Search Terms | (((pfna OR "c 1800") AND (fluorocarbon* OR fluorotelomer* OR polyfluoro* OR perfluoro* OR perfluorinated OR fluorinated OR pfas OR pfos OR pfoa)) OR "375-95-1" [rn] OR "2 2 3 3 4 4 5 5 6 6 7 7 8 8 9 9 9-heptadecafluorononanoic acid" OR "nonanoic acid 2 2 3 3 4 4 5 5 6 6 7 7 8 8 9 9 9-heptadecafluoro-" OR "nonanoic acid heptadecafluoro-" OR "perfluoro-n-nonanoic acid" OR "perfluorononan-1-oic acid" OR "perfluorononanoate" OR "perfluorononanoic acid" OR "perfluorononanonic acid" OR "perfluoropelargonic acid" OR "heptadecafluorononanoic acid") AND (ANEUPL [org] OR BIOSIS [org] OR CIS [org] OR DART [org] OR EMIC [org] OR EPIDEM [org] OR HEEP [org] OR HMTC [org] OR IPA [org] OR RISKLINE [org] OR MTGABS [org] OR NIOSH [org] OR NTIS [org] OR PESTAB [org] OR PPBIB [org]) AND NOT PubMed [org] AND NOT pubdart [org] |
| Literature Update and Additional PFNA Synonyms | @AND+@OR+(pfna+"c 1800"+fluorocarbon*+"2,2,3,3,4,4,5,5,6,6,7,7,8,8,9,9,9-heptadecafluorononanoic+acid"+"nonanoic+acid+2,2,3,3,4,4,5,5,6,6,7,7,8,8,9,9,9-heptadecafluoro-"+"nonanoic+acid+heptadecafluoro-"+"perfluoro-n-nonanoic+acid"+"perfluorononan-1-oic+acid"+perfluorononanoate+"perfluorononanoic+acid"+"perfluoropelargonic+acid"+"heptadecafluorononanoic+acid"+"Methyl-n1-Perfluorononanoic+acid"+"PFNA-n1CH3"+"EINECS 206-801-3"+"Heptadecafluornonansaeure"+"Heptadekafluornonansaeure"+"Perfluornonansaeure"+"Perfluorononanoic+acid (PFNA)"+"UNII-5830Z6S63M"+"perfluoro-n-nonanoic+acid"+"perfluorononan-1-oic+acid"+"perfluorononanoic+acid"+"Ammonium+Perfluorononanoate"+"Ammonium+perfluorononanoate"+"PFNA-H3N"+@TERM+@rn+375-95-1)+@RANGE+yr+2017+2018+2019+2020+2021+2022 |
| **TSCATS** | |
| Search Terms | "375-95-1" [rn] AND TSCATS[org] |
| Literature Update and Additional PFNA Synonyms | @TERM+@rn+375-95-1+@RANGE+yr+2017+2018+2019+2020+2021+2022 |

**Supplemental** **Figure 1. Study evaluation domains and ratings for epidemiology studies.**


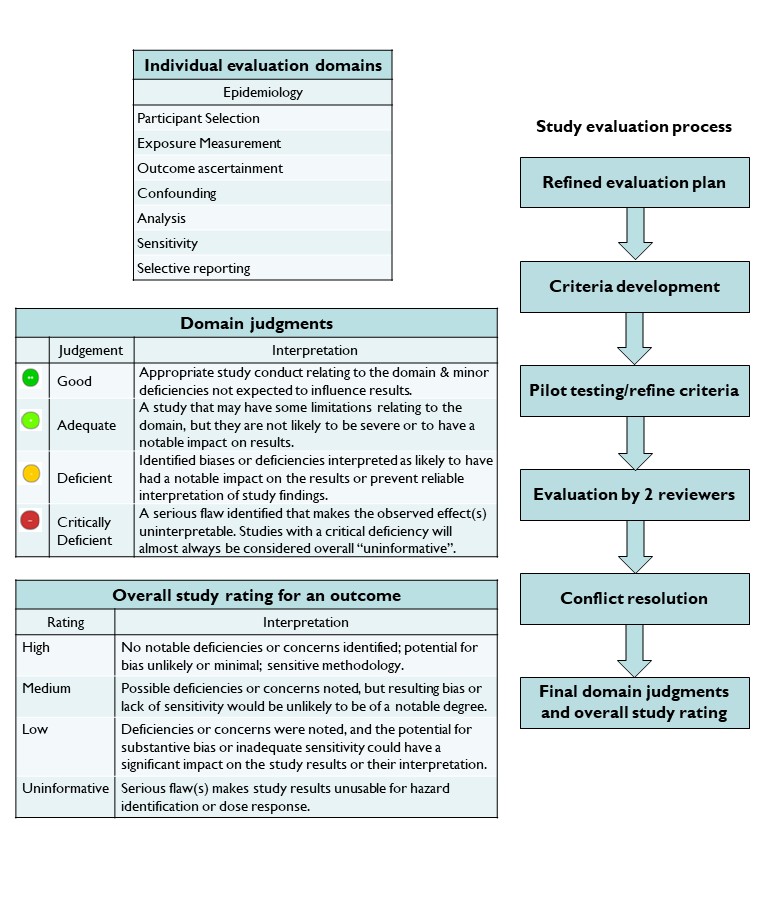


**Supplemental Figure 2. Summary of 41 BWT Studies Examined in the PFNA Study Evaluation and 27 Informative and Non-overlapping Studies Included in the Meta-analysis.**

**Supplemental Figure 3. Heat Map of 30 Non-overlapping Studies undergoing Study Evaluation**
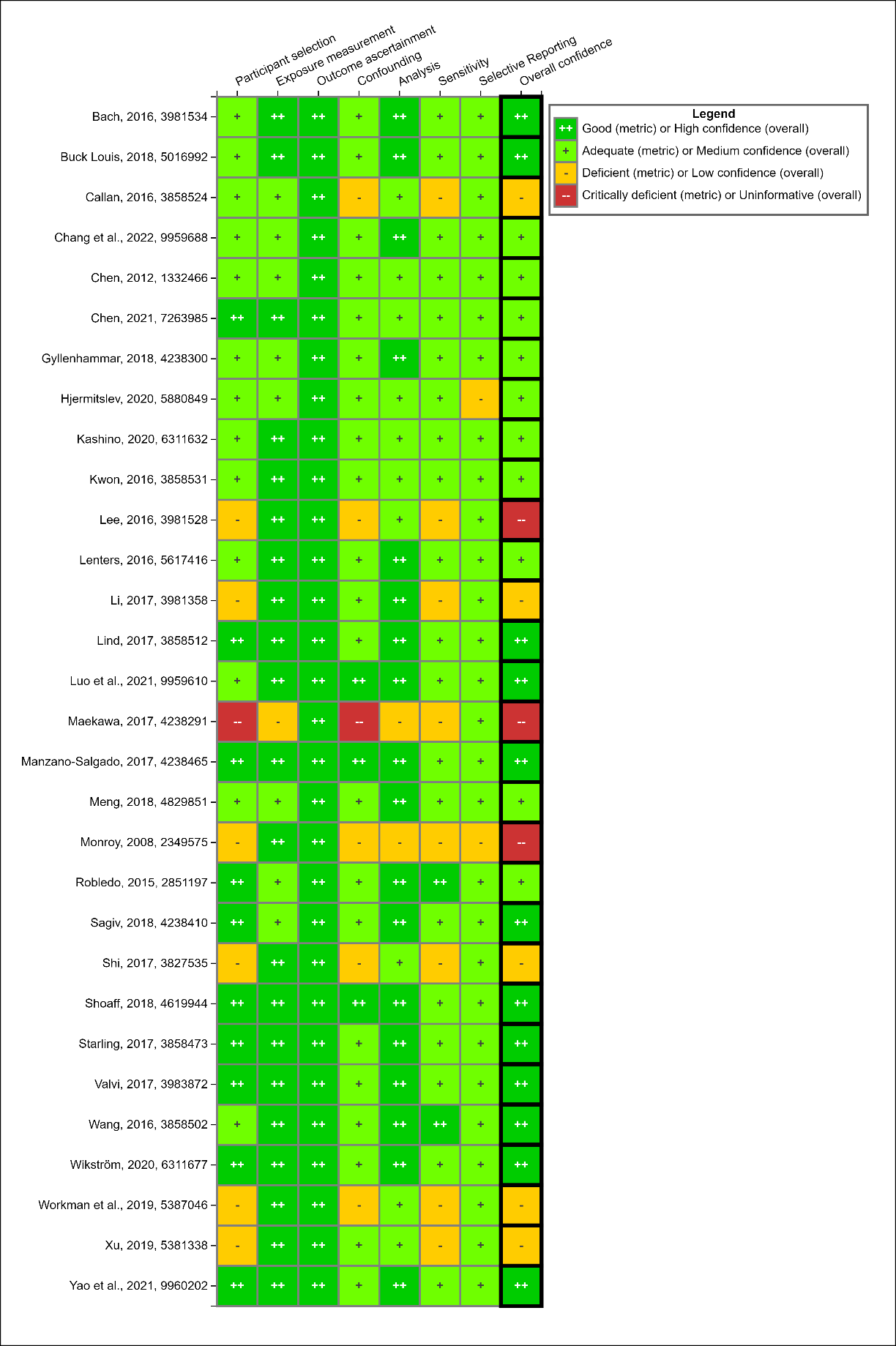


**Supplemental Table 3. Exposure biomarker sampling characteristics and classifications for 27 included studies.**

| **Study** | **Confidence** | **Sampling**  **Distribution (weeks)** | **Time Period** | **Sample Timing Strata** | **Notes** |
| --- | --- | --- | --- | --- | --- |
|  |  |  |  |  |  |
| **Bach et al., 2016** | High | 12 (9-20)  *Mode (Range)* | Trimesters 1, 2 | Early |  |
| **Buck Louis et al., 2018** | High | 10-13.9  *Range* | Trimester 1 | Early |  |
| **Chang et al., 2022** | Medium | 11.4 (9.6-12.6)  *Median (IQR)* | Trimesters 1, 2 | Early |  |
| **Chen et al., 2021** | Medium | 16.3 (13.9-20.4)  *Median (Range)* | Trimesters 1, 2 | Early | Authors provide additional data on specific sampling periods (Zhang, 2022). |
| **Hjermitslev et al., 2020** | Medium | 7-40  *Range* | Trimesters 1, 2, 3 | Early | Sampling predominantly occurred earlier in pregnancy, so assigned to *early* strata. Study authors report mean gestational sampling week=26.2 in 2010–2011 and all samples collected by end of week 13 in 2013–2015; 62% of samples were collected in 2013-2015 (Bonefeld-Jørgensen, 2022). |
| **Lind et al., 2017** | High | 10 (5-12)  *Median (Range)* | Trimester 1 | Early |  |
| **Manzano-Salgado et al., 2017** | High | 12.3 (5.6)  *Mean (SD)* | Trimester 1 | Early |  |
| **Meng et al., 2018** | Medium | 8  *Mean* | Trimesters 1, 2 | Early | The mean is reported in related publication by Liew et al. 2020. |
| **Robledo et al., 2015** | Medium | N/R | Preconception | Early |  |
| **Sagiv et al., 2018** | High | 9 (5-19)  *Median (Range*) | Trimesters 1, 2 | Early |  |
| **Wikström et al., 2020** | High | 10  *Median* | Trimesters 1, 2 | Early |  |
| **Callan et al., 2016** | Low | 33-40  *Range* | Trimester 3 | Mid/Late | Samples were taken two weeks before due date, which ranged from 35-42 weeks. |
| **Kashino et al., 2020** | Medium | 29  *Median* | Trimester 3 | Mid/Late |  |
| **Lenters et al., 2016** | Medium | 25.2  *Weighted mean of medians* | Trimesters 2, 3 | Mid/Late | Study authors reported country-specific medians: 33 weeks (Poland, 18%), 25 weeks (Greenland, 32%), 23 weeks (Ukraine, 49%). |
| **Luo et al., 2021** | High | 39.3  *Mean* | Trimester 3 | Mid/Late |  |
| **Shoaff et al., 2018** | High | N/R | Trimesters 2, 3, & At Delivery | Mid/Late | Study assigned to the late strata instead of post because only 5% of samples taken at delivery, and sensitivity analysis conducted by study authors found results robust to Trimester 2 only. |
| **Starling et al., 2017** | High | 27 (20-34)  *Median (Range)* | Trimesters 2, 3 | Mid/Late |  |
| **Valvi et al., 2017** | High | 34  *Point* | Trimester 3 | Mid/Late | All samples taken at gestational week 34. |
| **Wang et al., 2016** | High | N/R | Trimester 3 | Mid/Late |  |
| **Workman et al., 2019** | Low | 28.6 (14.3-39.6)  *Median (Range)* | Trimesters 2, 3 | Mid/Late |  |
| **Yao et al., 2021** | High | 39.4  *Mean* | Trimester 3 | Mid/Late |  |
| **Chen et al., 2012** | Medium | 39  *Median* | At Birth | Post |  |
| **Gyllenhammer et al., 2018** | Medium | 43 (37.9-46.1)  *Mean (Range)* | Post-Birth | Post | Samples taken 3 weeks after delivery; mean=40 weeks (range: 34.9-43.1) delivery date. |
| **Kwon et al., 2016** | Medium | 40 (N/R)  *Point* | At Delivery | Post |  |
| **Li et al., 2017** | Low | 39  *Mean* | At Delivery | Post |  |
| **Shi et al., 2017** | Low | 39.8 (4.2)  *Mean (SD)* | At Delivery | Post |  |
| **Xu et al., 2019** | Low | 39.4 (1.4)  *Mean (SD)* | At Delivery | Post |  |

**Supplemental Table 4. Comparison of overall effect estimated for different transformation assumptions in the Kwon et al. (2016) study*.**

| **Model** | **n** | **β** | **SE** | **95% CI** | **P** | **I^2^** | **P_q_** |
| --- | --- | --- | --- | --- | --- | --- | --- |
| Log Base e | 27 | -32.9 | 7.2 | -47.0, -18.7 | 0.0 | 35.9 | 0.05 |
| Log Base 2 | 27 | -33.0 | 7.4 | -47.5, -18.6 | 0.0 | 36.5 | 0.04 |
| Log Base 10 | 27 | -30.6 | 6.6 | -43.6, -17.6 | 0.0 | 32.3 | 0.09 |

Abbreviations: n: number of studies; β: beta coefficient for pooled estimate; SE: standard error; CI: confidence interval; P = p-value (alpha = 0.05) for the hypothesis test of whether the estimate is equal to zero; I^2^ = percentage of variability due to heterogeneity; P_q_ = p-value (alpha = 0.05) for the hypothesis test of whether the between-study variation is equal to zero.

*The study Kwon et al., 2016 reported results on the log-scale, but did not specify the base of the log. The main analysis assumes base e (i.e., natural log or ln) vs. a log base of 2 or 10 assumption here.

**Supplemental Table 5. Random vs. fixed effect model results for the overall meta-analysis (n = 27 studies)*.**

| **Model** | **β** | **SE** | **95% CI** | **P** | **I^2^** | **P_q_** |
| --- | --- | --- | --- | --- | --- | --- |
| Random | -32.9 | 7.2 | -47.0, -18.7 | 0 | 35.93 | 0.05 |
| Fixed | -26.8 | 5.1 | -36.8, -16.8 | 0 | 32.58 | 0.05 |

Abbreviations: β: beta coefficient i.e., the pooled estimate; SE: standard error; CI: confidence interval; P-Value: for the hypothesis test of whether β is equal to zero (significance level, alpha = 0.05); I^2^ = percentage of variability due to heterogeneity; P**_q_**: for the hypothesis test of whether the between-study variation is equal to zero, i.e., p_Q_ (significance level, alpha = 0.05).

*Since our analysis employed inverse-variance weights, the difference in magnitude between the random and fixed effects estimate is indicative of possible study heterogeneity. This is because the weights (w) used in random and fixed effect models are w_R_ = 1 / (v + t) and w_F_ = 1 / v, respectively, where v represents within-study variance and t represents between-study heterogeneity (Borenstein et al., 2010). So, if there were no heterogeneity, i.e., t = 0, then the weights in a random effects model would simplify to w_R_ = 1 / (v + 0) = 1 / v = w_F_, and we would expect the random effects model to yield the same pooled effect as the fixed. Furthermore, the results of the hypothesis test for whether study heterogeneity equaled zero yielded nearly statistically significant results.
